# Supplementary material for: Variations in terrestrial arthropod DNA metabarcoding methods recovers robust beta diversity but variable richness and site indicators
Source: Sci Rep. 2019 Dec 3;9:18218. doi: 10.1038/s41598-019-54532-0 (PMC6890670; doi:10.1038/s41598-019-54532-0)
Supplement: Supplementary file 1 — Supplementary Information [file 41598_2019_54532_MOESM1_ESM.pdf]

Supporting Information

Variations in terrestrial arthropod DNA metabarcoding methods recovers robust beta diversity but variable richness and site indicators

Teresita M. Porter[1,2*], Dave M. Morris[3], Nathan Basiliko[4], Mehrdad Hajibabaei[2], Daniel Doucet[1], Susan Bowman[1], Erik J.S. Emilson[1], Caroline E. Emilson[1], Derek Chartrand[1], Kerrie Wainio-Keizer[1], Armand Séguin[5], Lisa Venier[1]

[1]Great Lakes Forestry Centre, Natural Resources Canada, Sault Ste. Marie, ON, P6A 2E5

[2]Biodiversity Institute of Ontario, Centre for Biodiversity Genomics & Integrative Biology, University of Guelph, Guelph, ON, N1G 2W1

[3]Ministry of Natural Resources and Forestry, Centre for Northern Forest Ecosystem Research, Thunder Bay, ON, P7E 2V6

[4]Laurentian University Department of Biology and the Vale Living with Lakes Centre, Sudbury, ON, P3E 2C6

[5]Laurentian Forestry Centre, Natural Resources Canada, Québec, QC, G1V 4C7

Table S1. Read counts for all taxa

	Marker		
Data	BE	F230R_modN	BE+F230R_modN
Raw x 2	22,742,760	18,442,391	41,185,151
Paired	17,941,208	17,354,156	35,295,364
Primer-trimmed	16,184,320	17,011,213	33,195,533

Table S2. ESV counts for all taxa

	Marker		
Data	BE	F230R_modN	BE+F230R_modN
ESVs	50,857	16,769	67,626
Reads in ESVs	8,269,741	11,292,505	19,562,246
Proportion of raw reads (%)	36.4	61.2	47.5

Table S3. Arthropoda ESV counts

	Marker		
Data	BE	F230R_modN	BE+F230R_modN
Arthropoda ESVs	775	2,823	3,598
Reads in Arthropoda ESVs	294,070	2,398,638	2,692,708
Proportion of raw reads (%)	1.3	13	6.5

Fig S1. Original taxonomic distribution of sequenced reads. For clarity, only results from the top 10 most common phyla by ESVs or reads are shown. ESV and read numbers are shown for the top three groups.

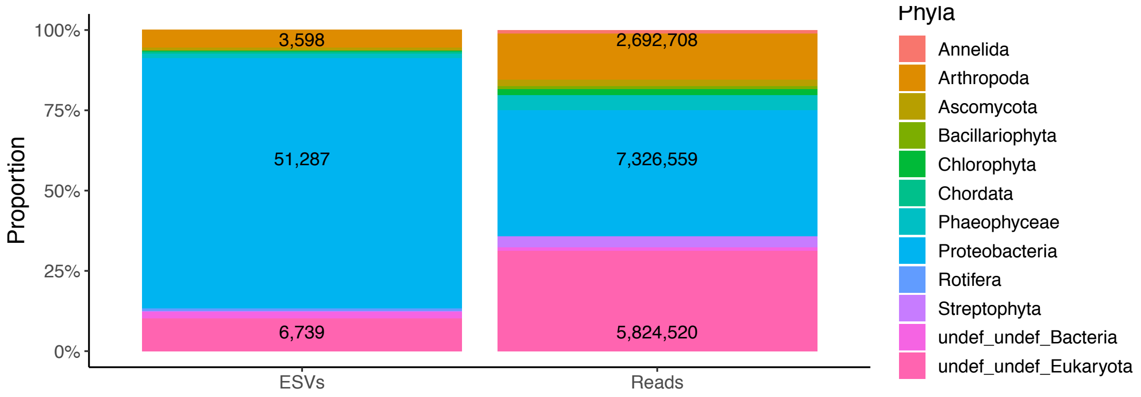

Fig S2: A small proportion of arthropoda could be confidently identified using the COI classifier v3 reference database. Results are shown at the species, genus, and family ranks where minimum bootstrap support cutoffs were used to filter for confidently identified taxa.

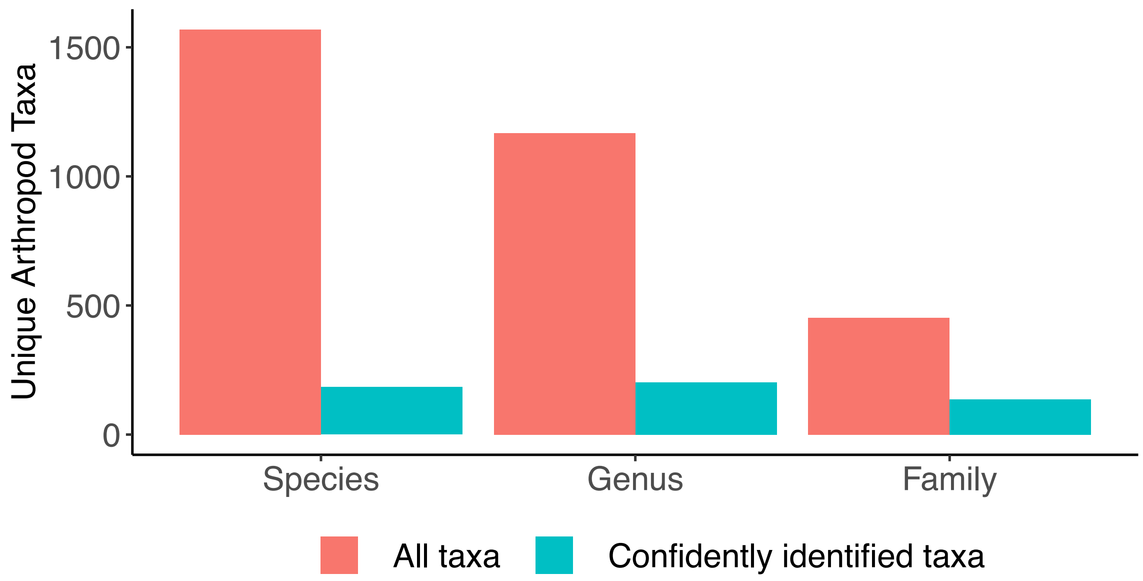

Fig S3. Arthropoda sequencing is saturated. Rarefaction curves from the Island lake site (ILC) (top row) and the Nimitz site (NZC) (bottom row) are shown. Reads are plotted against exact sequence variants (ESVs) for each set of experiments: 1C1E (one soil core, one DNA extraction), 1C3E (one soil core, three pooled DNA extractions), XC3E (2-15 pooled soil cores, three pooled DNA extractions). Curves are colored according to the layer they were sampled from: bryophyte layer (green), organic horizon (blue), mineral horizon (orange). The vertical guide line indicates the library size at the 15th percentile.

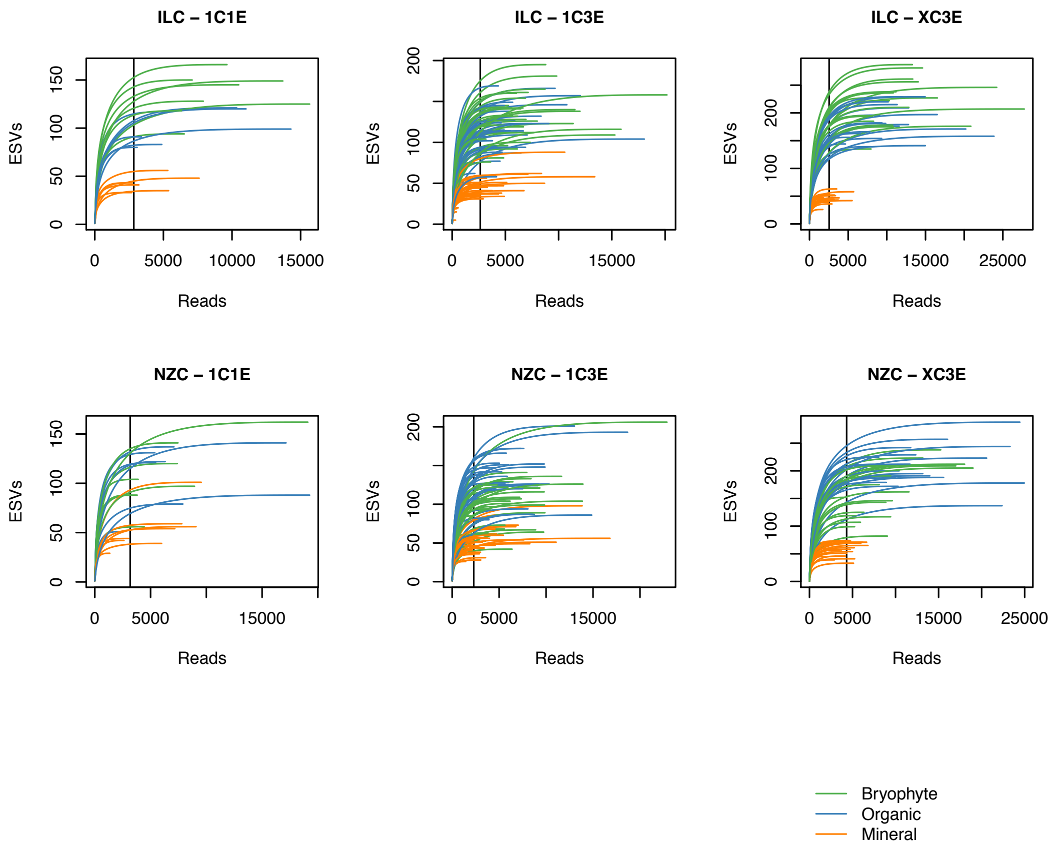

Fig S4. Many unique ESVs are recovered from each soil layer, especially from the bryophyte and organic layers. Based on normalized library sizes. Data pooled across all samples and methods.

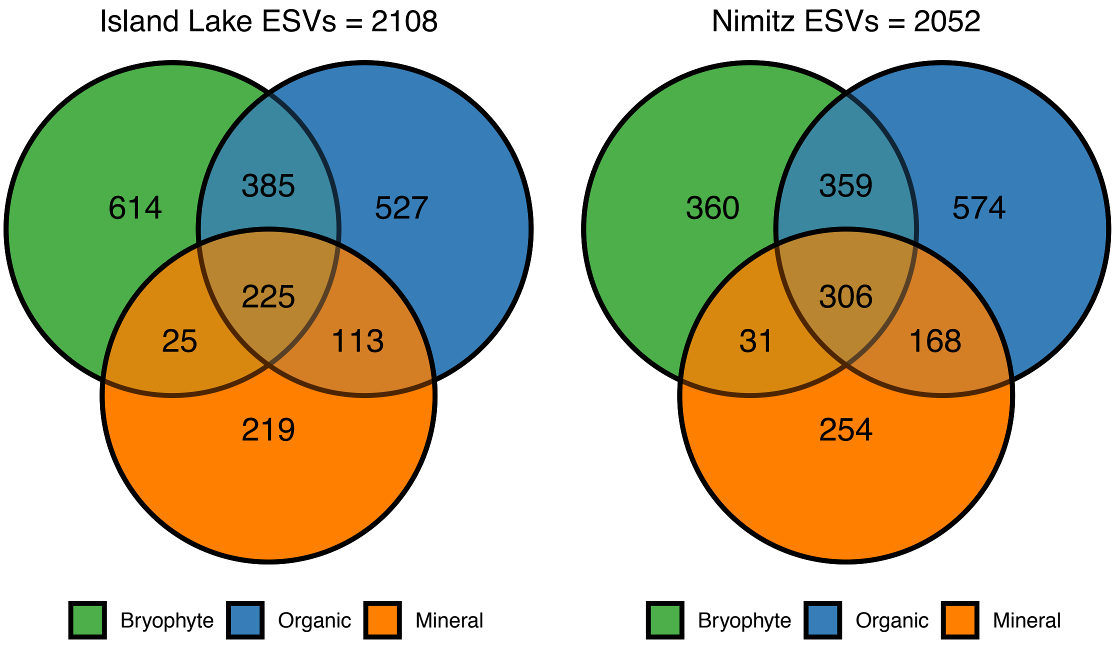

Fig S5. Arthropod richness increases varies little when more DNA extractions are performed. This figure is a supplement to Fig 1C in the main text. No significant differences found in the richness across sites or number of extractions. Significant differences in richness found across some soil layers (bryophyte compared with mineral, p-value = 4.1e-10; organic compared with mineral, p-value = 3.0e-10) but not when comparing the bryophyte-organic layers.

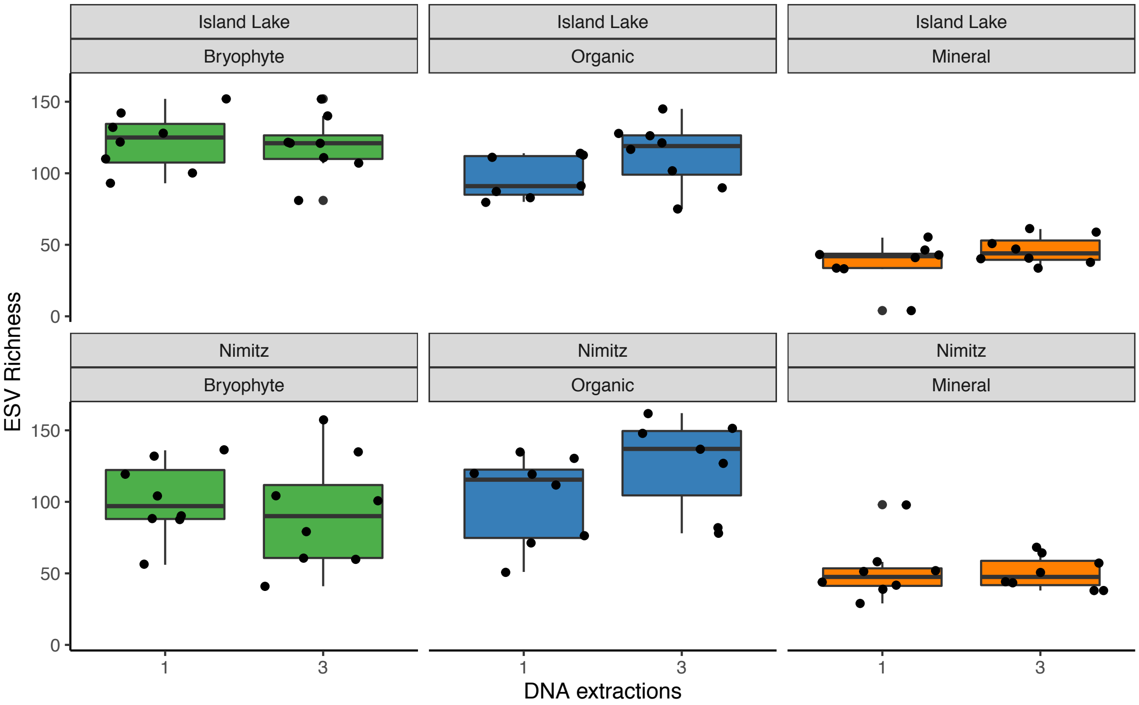

Fig S6. Site indicators based on exact sequence variants distinguishes among two sites. Although indicator taxa summarized to higher taxonomic ranks appear similar across sites due to our inability to confidently identify ESVs (Fig 3), sites are clearly distinguished using ESVs sampled using a variety of methods. Method abbreviations: 1C1E – one core, 1 DNA extraction; 1C3E – one core, 3 pooled DNA extractions; 2C3E – 2 pooled cores, 3 pooled DNA extractions; 4C3E – 4 pooled cores, 3 pooled DNA extractions; 6C3E – 6 pooled cores, 3 pooled DNA extractions; 8C3E – 8 pooled cores, 3 pooled DNA extractions; 915C3E – 9-15 pooled cores, 3 pooled DNA extractions. Samples from each method were subsampled down to 4 for a balanced design. For each sample, results from 3 layers (bryophyte, organic, mineral) were combined (n = 12 for each method).

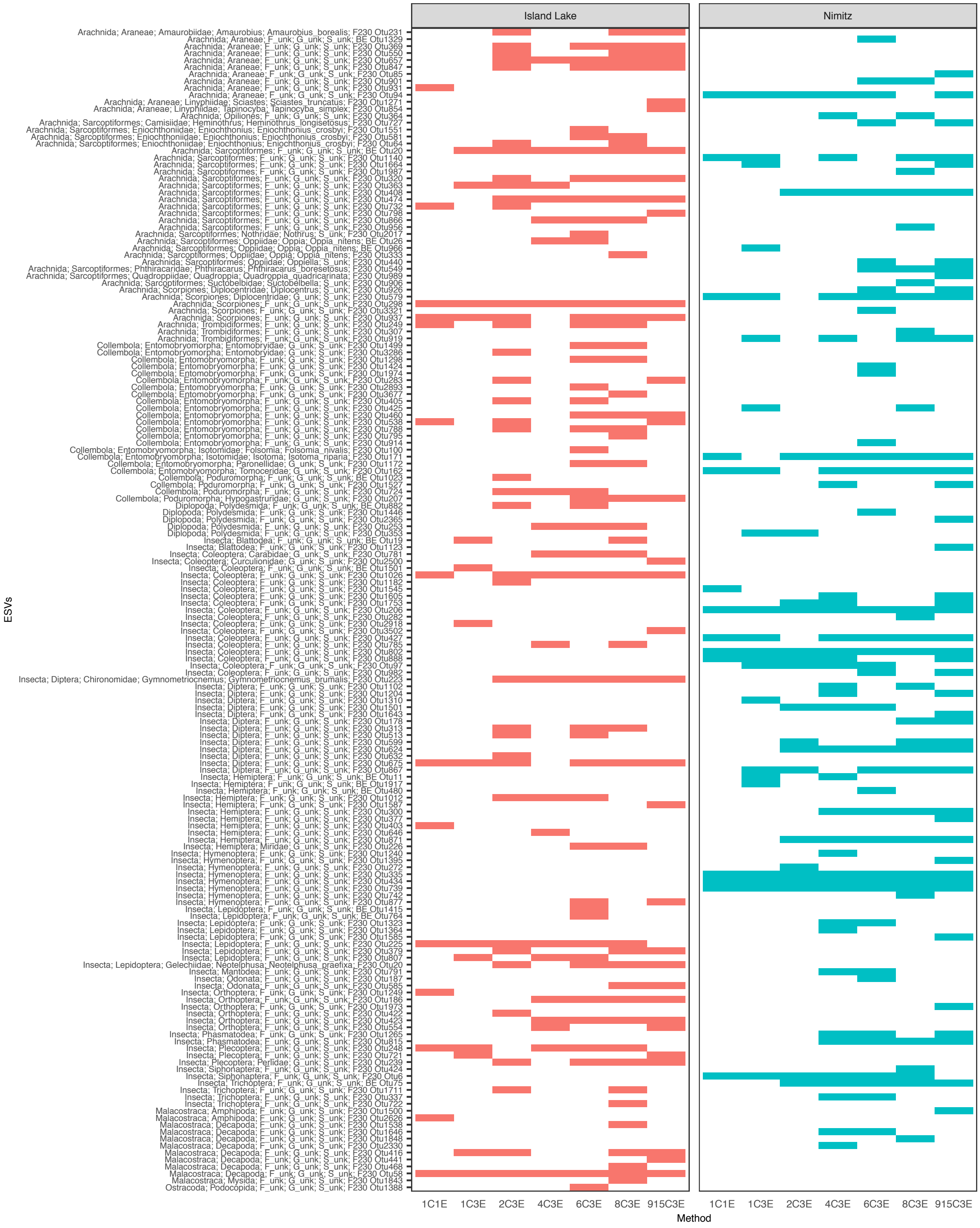

Island Lake
Nimitz
ESVs
Arachnida; Araneae; Amaurobiidae; Amaurobius; Amaurobius_borealis; F230 Otu231
Arachnida; Araneae; F_unk; G_unk; S_unk; BE Otu1329
Arachnida; Araneae; F_unk; G_unk; S_unk; F230 Otu369
Arachnida; Araneae; F_unk; G_unk; S_unk; F230 Otu550
Arachnida; Araneae; F_unk; G_unk; S_unk; F230 Otu657
Arachnida; Araneae; F_unk; G_unk; S_unk; F230 Otu847
Arachnida; Araneae; F_unk; G_unk; S_unk; F230 Otu85
Arachnida; Araneae; F_unk; G_unk; S_unk; F230 Otu901
Arachnida; Araneae; F_unk; G_unk; S_unk; F230 Otu931
Arachnida; Araneae; F_unk; G_unk; S_unk; F230 Otu94
Arachnida; Araneae; Linyphiidae; Sciastes; Sciastes_truncatus; F230 Otu1271
Arachnida; Araneae; Linyphiidae; Tapinocyba; Tapinocyba_simplex; F230 Otu854
Arachnida; Opiliones; F_unk; G_unk; S_unk; F230 Otu364
Arachnida; Sarcoptiformes; Camisiidae; Heminothrus; Heminothrus_longisetosus; F230 Otu727
Arachnida; Sarcoptiformes; Eniochthoniidae; Eniochthonius; Eniochthonius_crosbyi; F230 Otu1551
Arachnida; Sarcoptiformes; Eniochthoniidae; Eniochthonius; Eniochthonius_crosbyi; F230 Otu581
Arachnida; Sarcoptiformes; Eniochthoniidae; Eniochthonius; Eniochthonius_crosbyi; F230 Otu64
Arachnida; Sarcoptiformes; F_unk; G_unk; S_unk; BE Otu20
Arachnida; Sarcoptiformes; F_unk; G_unk; S_unk; F230 Otu1140
Arachnida; Sarcoptiformes; F_unk; G_unk; S_unk; F230 Otu1664
Arachnida; Sarcoptiformes; F_unk; G_unk; S_unk; F230 Otu1987
Arachnida; Sarcoptiformes; F_unk; G_unk; S_unk; F230 Otu320
Arachnida; Sarcoptiformes; F_unk; G_unk; S_unk; F230 Otu363
Arachnida; Sarcoptiformes; F_unk; G_unk; S_unk; F230 Otu408
Arachnida; Sarcoptiformes; F_unk; G_unk; S_unk; F230 Otu474
Arachnida; Sarcoptiformes; F_unk; G_unk; S_unk; F230 Otu732
Arachnida; Sarcoptiformes; F_unk; G_unk; S_unk; F230 Otu798
Arachnida; Sarcoptiformes; F_unk; G_unk; S_unk; F230 Otu866
Arachnida; Sarcoptiformes; F_unk; G_unk; S_unk; F230 Otu956
Arachnida; Sarcoptiformes; Nothridae; Nothrus; S_unk; F230 Otu2017
Arachnida; Sarcoptiformes; Oppiidae; Oppia; Oppia_nitens; BE Otu26
Arachnida; Sarcoptiformes; Oppiidae; Oppia; Oppia_nitens; BE Otu966
Arachnida; Sarcoptiformes; Oppiidae; Oppia; Oppia_nitens; F230 Otu333
Arachnida; Sarcoptiformes; Oppiidae; Oppiella; S_unk; F230 Otu440
Arachnida; Sarcoptiformes; Phthiracaridae; Phthiracarus; Phthiracarus_boresetosus; F230 Otu549
Arachnida; Sarcoptiformes; Quadroppiidae; Quadroppia; Quadroppia_quadricarinata; F230 Otu989
Arachnida; Sarcoptiformes; Suctobelbidae; Suctobelbella; S_unk; F230 Otu906
Arachnida; Scorpiones; Diplocentridae; Diplocentrus; S_unk; F230 Otu926
Arachnida; Scorpiones; Diplocentridae; G_unk; S_unk; F230 Otu579
Arachnida; Scorpiones; F_unk; G_unk; S_unk; F230 Otu298
Arachnida; Scorpiones; F_unk; G_unk; S_unk; F230 Otu3321
Arachnida; Scorpiones; F_unk; G_unk; S_unk; F230 Otu937
Arachnida; Trombidiformes; F_unk; G_unk; S_unk; F230 Otu249
Arachnida; Trombidiformes; F_unk; G_unk; S_unk; F230 Otu307
Arachnida; Trombidiformes; F_unk; G_unk; S_unk; F230 Otu919
Collembola; Entomobryomorpha; Entomobryidae; G_unk; S_unk; F230 Otu1499
Collembola; Entomobryomorpha; Entomobryidae; G_unk; S_unk; F230 Otu3286
Collembola; Entomobryomorpha; F_unk; G_unk; S_unk; F230 Otu1298
Collembola; Entomobryomorpha; F_unk; G_unk; S_unk; F230 Otu1424
Collembola; Entomobryomorpha; F_unk; G_unk; S_unk; F230 Otu1974
Collembola; Entomobryomorpha; F_unk; G_unk; S_unk; F230 Otu283
Collembola; Entomobryomorpha; F_unk; G_unk; S_unk; F230 Otu2893
Collembola; Entomobryomorpha; F_unk; G_unk; S_unk; F230 Otu3677
Collembola; Entomobryomorpha; F_unk; G_unk; S_unk; F230 Otu405
Collembola; Entomobryomorpha; F_unk; G_unk; S_unk; F230 Otu425
Collembola; Entomobryomorpha; F_unk; G_unk; S_unk; F230 Otu460
Collembola; Entomobryomorpha; F_unk; G_unk; S_unk; F230 Otu538
Collembola; Entomobryomorpha; F_unk; G_unk; S_unk; F230 Otu788
Collembola; Entomobryomorpha; F_unk; G_unk; S_unk; F230 Otu795
Collembola; Entomobryomorpha; F_unk; G_unk; S_unk; F230 Otu914
Collembola; Entomobryomorpha; Isotomidae; Folsomia; Folsomia_nivalis; F230 Otu100
Collembola; Entomobryomorpha; Isotomidae; Isotoma; Isotoma_riparia; F230 Otu171
Collembola; Entomobryomorpha; Paronellidae; G_unk; S_unk; F230 Otu1172
Collembola; Entomobryomorpha; Tomoceridae; G_unk; S_unk; F230 Otu162
Collembola; Poduromorpha; F_unk; G_unk; S_unk; BE Otu1023
Collembola; Poduromorpha; F_unk; G_unk; S_unk; F230 Otu1527
Collembola; Poduromorpha; F_unk; G_unk; S_unk; F230 Otu724
Collembola; Poduromorpha; Hypogastruridae; G_unk; S_unk; F230 Otu207
Diplopoda; Polydesmida; F_unk; G_unk; S_unk; BE Otu882
Diplopoda; Polydesmida; F_unk; G_unk; S_unk; F230 Otu1446
Diplopoda; Polydesmida; F_unk; G_unk; S_unk; F230 Otu2365
Diplopoda; Polydesmida; F_unk; G_unk; S_unk; F230 Otu253
Diplopoda; Polydesmida; F_unk; G_unk; S_unk; F230 Otu353
Insecta; Blattodea; F_unk; G_unk; S_unk; BE Otu19
Insecta; Blattodea; F_unk; G_unk; S_unk; F230 Otu1123
Insecta; Coleoptera; Carabidae; G_unk; S_unk; F230 Otu781
Insecta; Coleoptera; Curculionidae; G_unk; S_unk; F230 Otu2500
Insecta; Coleoptera; F_unk; G_unk; S_unk; BE Otu1501
Insecta; Coleoptera; F_unk; G_unk; S_unk; F230 Otu1026
Insecta; Coleoptera; F_unk; G_unk; S_unk; F230 Otu1182
Insecta; Coleoptera; F_unk; G_unk; S_unk; F230 Otu1545
Insecta; Coleoptera; F_unk; G_unk; S_unk; F230 Otu1605
Insecta; Coleoptera; F_unk; G_unk; S_unk; F230 Otu1753
Insecta; Coleoptera; F_unk; G_unk; S_unk; F230 Otu206
Insecta; Coleoptera; F_unk; G_unk; S_unk; F230 Otu282
Insecta; Coleoptera; F_unk; G_unk; S_unk; F230 Otu2918
Insecta; Coleoptera; F_unk; G_unk; S_unk; F230 Otu3502
Insecta; Coleoptera; F_unk; G_unk; S_unk; F230 Otu427
Insecta; Coleoptera; F_unk; G_unk; S_unk; F230 Otu785
Insecta; Coleoptera; F_unk; G_unk; S_unk; F230 Otu802
Insecta; Coleoptera; F_unk; G_unk; S_unk; F230 Otu888
Insecta; Coleoptera; F_unk; G_unk; S_unk; F230 Otu97
Insecta; Coleoptera; F_unk; G_unk; S_unk; F230 Otu982
Insecta; Diptera; Chironomidae; Gymnometriocnemus; Gymnometriocnemus_brumalis; F230 Otu223
Insecta; Diptera; F_unk; G_unk; S_unk; F230 Otu1102
Insecta; Diptera; F_unk; G_unk; S_unk; F230 Otu1204
Insecta; Diptera; F_unk; G_unk; S_unk; F230 Otu1310
Insecta; Diptera; F_unk; G_unk; S_unk; F230 Otu1501
Insecta; Diptera; F_unk; G_unk; S_unk; F230 Otu1643
Insecta; Diptera; F_unk; G_unk; S_unk; F230 Otu178
Insecta; Diptera; F_unk; G_unk; S_unk; F230 Otu313
Insecta; Diptera; F_unk; G_unk; S_unk; F230 Otu513
Insecta; Diptera; F_unk; G_unk; S_unk; F230 Otu599
Insecta; Diptera; F_unk; G_unk; S_unk; F230 Otu624
Insecta; Diptera; F_unk; G_unk; S_unk; F230 Otu632
Insecta; Diptera; F_unk; G_unk; S_unk; F230 Otu675
Insecta; Diptera; F_unk; G_unk; S_unk; F230 Otu867
Insecta; Hemiptera; F_unk; G_unk; S_unk; BE Otu11
Insecta; Hemiptera; F_unk; G_unk; S_unk; BE Otu1917
Insecta; Hemiptera; F_unk; G_unk; S_unk; BE Otu480
Insecta; Hemiptera; F_unk; G_unk; S_unk; F230 Otu1012
Insecta; Hemiptera; F_unk; G_unk; S_unk; F230 Otu1587
Insecta; Hemiptera; F_unk; G_unk; S_unk; F230 Otu300
Insecta; Hemiptera; F_unk; G_unk; S_unk; F230 Otu377
Insecta; Hemiptera; F_unk; G_unk; S_unk; F230 Otu403
Insecta; Hemiptera; F_unk; G_unk; S_unk; F230 Otu646
Insecta; Hemiptera; F_unk; G_unk; S_unk; F230 Otu871
Insecta; Hemiptera; Miridae; G_unk; S_unk; F230 Otu226
Insecta; Hymenoptera; F_unk; G_unk; S_unk; F230 Otu1240
Insecta; Hymenoptera; F_unk; G_unk; S_unk; F230 Otu1395
Insecta; Hymenoptera; F_unk; G_unk; S_unk; F230 Otu272
Insecta; Hymenoptera; F_unk; G_unk; S_unk; F230 Otu335
Insecta; Hymenoptera; F_unk; G_unk; S_unk; F230 Otu434
Insecta; Hymenoptera; F_unk; G_unk; S_unk; F230 Otu739
Insecta; Hymenoptera; F_unk; G_unk; S_unk; F230 Otu742
Insecta; Hymenoptera; F_unk; G_unk; S_unk; F230 Otu877
Insecta; Lepidoptera; F_unk; G_unk; S_unk; BE Otu1415
Insecta; Lepidoptera; F_unk; G_unk; S_unk; BE Otu764
Insecta; Lepidoptera; F_unk; G_unk; S_unk; F230 Otu1323
Insecta; Lepidoptera; F_unk; G_unk; S_unk; F230 Otu1364
Insecta; Lepidoptera; F_unk; G_unk; S_unk; F230 Otu1585
Insecta; Lepidoptera; F_unk; G_unk; S_unk; F230 Otu225
Insecta; Lepidoptera; F_unk; G_unk; S_unk; F230 Otu379
Insecta; Lepidoptera; F_unk; G_unk; S_unk; F230 Otu807
Insecta; Lepidoptera; Gelechiidae; Neotelphusa; Neotelphusa_praefixa; F230 Otu20
Insecta; Mantodea; F_unk; G_unk; S_unk; F230 Otu791
Insecta; Odonata; F_unk; G_unk; S_unk; F230 Otu187
Insecta; Odonata; F_unk; G_unk; S_unk; F230 Otu585
Insecta; Orthoptera; F_unk; G_unk; S_unk; F230 Otu1249
Insecta; Orthoptera; F_unk; G_unk; S_unk; F230 Otu186
Insecta; Orthoptera; F_unk; G_unk; S_unk; F230 Otu1973
Insecta; Orthoptera; F_unk; G_unk; S_unk; F230 Otu422
Insecta; Orthoptera; F_unk; G_unk; S_unk; F230 Otu423
Insecta; Orthoptera; F_unk; G_unk; S_unk; F230 Otu554
Insecta; Phasmatodea; F_unk; G_unk; S_unk; F230 Otu1265
Insecta; Phasmatodea; F_unk; G_unk; S_unk; F230 Otu815
Insecta; Plecoptera; F_unk; G_unk; S_unk; F230 Otu248
Insecta; Plecoptera; F_unk; G_unk; S_unk; F230 Otu721
Insecta; Plecoptera; Perlidae; G_unk; S_unk; F230 Otu239
Insecta; Siphonaptera; F_unk; G_unk; S_unk; F230 Otu424
Insecta; Siphonaptera; F_unk; G_unk; S_unk; F230 Otu6
Insecta; Trichoptera; F_unk; G_unk; S_unk; BE Otu75
Insecta; Trichoptera; F_unk; G_unk; S_unk; F230 Otu1711
Insecta; Trichoptera; F_unk; G_unk; S_unk; F230 Otu337
Insecta; Trichoptera; F_unk; G_unk; S_unk; F230 Otu722
Malacostraca; Amphipoda; F_unk; G_unk; S_unk; F230 Otu1500
Malacostraca; Amphipoda; F_unk; G_unk; S_unk; F230 Otu2626
Malacostraca; Decapoda; F_unk; G_unk; S_unk; F230 Otu1538
Malacostraca; Decapoda; F_unk; G_unk; S_unk; F230 Otu1646
Malacostraca; Decapoda; F_unk; G_unk; S_unk; F230 Otu1848
Malacostraca; Decapoda; F_unk; G_unk; S_unk; F230 Otu2330
Malacostraca; Decapoda; F_unk; G_unk; S_unk; F230 Otu416
Malacostraca; Decapoda; F_unk; G_unk; S_unk; F230 Otu441
Malacostraca; Decapoda; F_unk; G_unk; S_unk; F230 Otu468
Malacostraca; Decapoda; F_unk; G_unk; S_unk; F230 Otu58
Malacostraca; Mysida; F_unk; G_unk; S_unk; F230 Otu1843
Ostracoda; Podocopida; F_unk; G_unk; S_unk; F230 Otu1388
1C1E
1C3E
2C3E
4C3E
6C3E
8C3E
915C3E
1C1E
1C3E
2C3E
4C3E
6C3E
8C3E
915C3E
Method

Fig S7. Similar site indicator taxa are recovered whether 1 to 15 pooled samples are used. Heat trees comprised of all the site indicator ESVs for each set of pooled samples. The data was pooled across both sites, then the 1C3E samples were randomly subsampled down to 4 samples per layer for a balanced comparison with the pooled samples from XC3E. In each heat tree, color indicates the number of samples where each taxon was detected; text and node size represent the number of site indicator ESVs in each taxon. To improve readability, labels have been added only to nodes present in at least half the plotted samples. Taxa that could not be confidently identified are indicated by an asterisk (*).

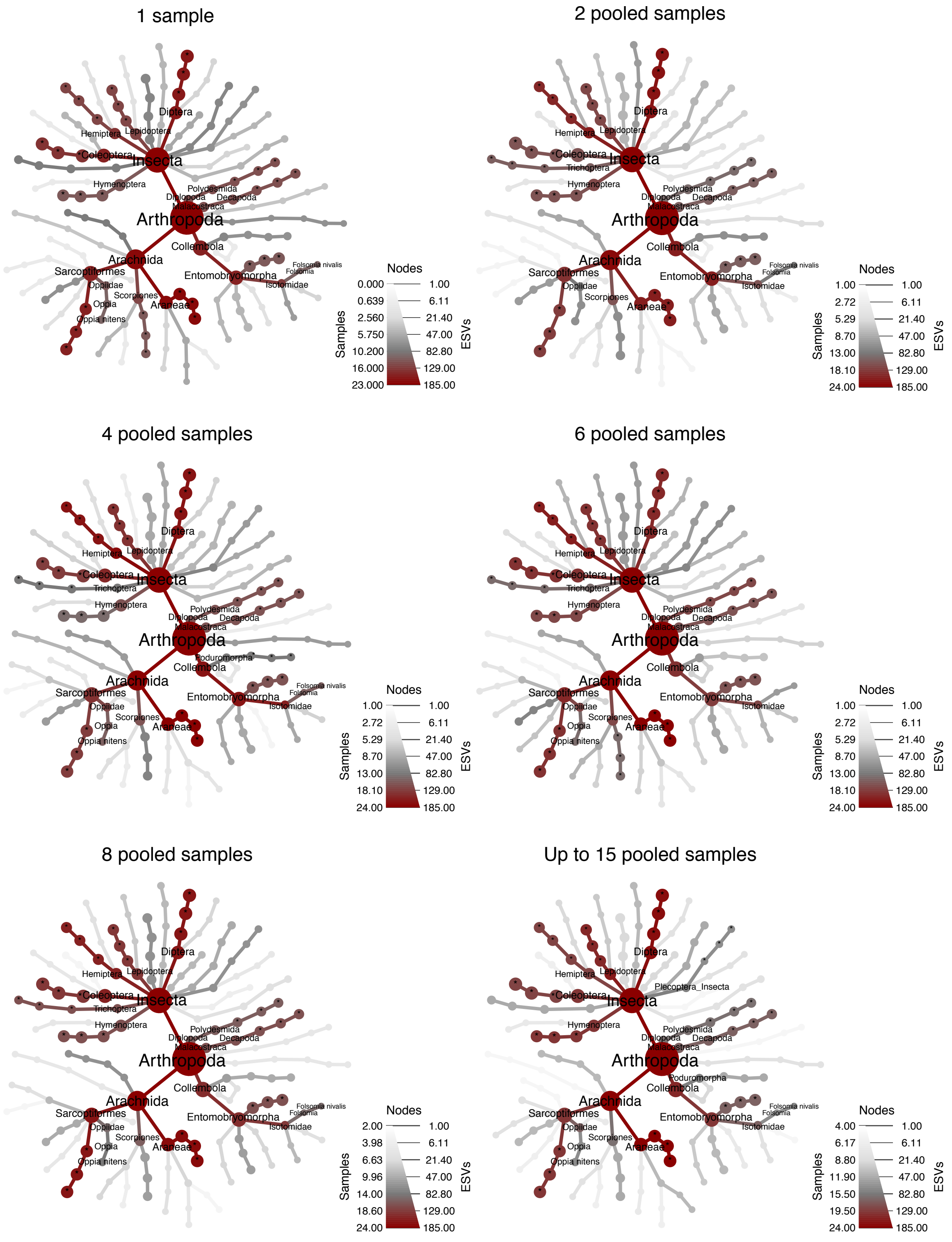

1 sample
Diptera
Hemiptera
Lepidoptera
Coleoptera
Insecta
Hymenoptera
Polydesmida
Diplopoda
Decapoda
Malacostraca
Arthropoda
Collembola
Arachnida
Sarcoptiformes
Oppiidae
Scorpiones
Oppia
Araneae
Oppia nitens
Entomobryomorpha
Folsomia nivalis
Folsomia
Isotomidae
Nodes
Samples
0.000
0.639
2.560
5.750
10.200
16.000
23.000
ESVs
1.00
6.11
21.40
47.00
82.80
129.00
185.00
2 pooled samples
Diptera
Hemiptera
Lepidoptera
Coleoptera
Insecta
Trichoptera
Hymenoptera
Polydesmida
Diplopoda
Decapoda
Malacostraca
Arthropoda
Collembola
Arachnida
Sarcoptiformes
Oppiidae
Scorpiones
Araneae
Entomobryomorpha
Folsomia nivalis
Folsomia
Isotomidae
Nodes
Samples
1.00
2.72
5.29
8.70
13.00
18.10
24.00
ESVs
1.00
6.11
21.40
47.00
82.80
129.00
185.00
4 pooled samples
Diptera
Hemiptera
Lepidoptera
Coleoptera
Insecta
Trichoptera
Hymenoptera
Polydesmida
Diplopoda
Decapoda
Malacostraca
Arthropoda
Poduromorpha
Collembola
Arachnida
Sarcoptiformes
Oppiidae
Scorpiones
Oppia
Araneae
Oppia nitens
Entomobryomorpha
Folsomia nivalis
Folsomia
Isotomidae
Nodes
Samples
1.00
2.72
5.29
8.70
13.00
18.10
24.00
ESVs
1.00
6.11
21.40
47.00
82.80
129.00
185.00
6 pooled samples
Diptera
Hemiptera
Lepidoptera
Coleoptera
Insecta
Trichoptera
Hymenoptera
Polydesmida
Diplopoda
Decapoda
Malacostraca
Arthropoda
Collembola
Arachnida
Sarcoptiformes
Oppiidae
Scorpiones
Oppia
Araneae
Oppia nitens
Entomobryomorpha
Isotomidae
Nodes
Samples
1.00
2.72
5.29
8.70
13.00
18.10
24.00
ESVs
1.00
6.11
21.40
47.00
82.80
129.00
185.00
8 pooled samples
Diptera
Hemiptera
Lepidoptera
Coleoptera
Insecta
Trichoptera
Hymenoptera
Polydesmida
Diplopoda
Decapoda
Malacostraca
Arthropoda
Collembola
Arachnida
Sarcoptiformes
Oppiidae
Scorpiones
Oppia
Araneae
Oppia nitens
Entomobryomorpha
Folsomia nivalis
Folsomia
Isotomidae
Nodes
Samples
2.00
3.98
6.63
9.96
14.00
18.60
24.00
ESVs
1.00
6.11
21.40
47.00
82.80
129.00
185.00
Up to 15 pooled samples
Diptera
Hemiptera
Lepidoptera
Plecoptera_Insecta
Coleoptera
Insecta
Hymenoptera
Polydesmida
Diplopoda
Decapoda
Malacostraca
Arthropoda
Poduromorpha
Collembola
Arachnida
Sarcoptiformes
Oppiidae
Scorpiones
Oppia
Araneae
Oppia nitens
Entomobryomorpha
Folsomia nivalis
Folsomia
Isotomidae
Nodes
Samples
4.00
6.17
8.80
11.90
15.50
19.50
24.00
ESVs
1.00
6.11
21.40
47.00
82.80
129.00
185.00

Fig S8. Similar site indicator taxa are recovered whether 1 or 3 DNA extractions are used. Heat trees comprised of all the site indicator ESVs detected when 1 or 3 DNA extractions are used. The data was pooled across both sites and the same 4 grid coordinates per layer were compared across the 1C1E and 1C3E methods for a balanced comparison. In each heat tree, color indicates the number of samples where each taxon was detected; text and node size represent the number of site indicator ESVs in each taxon. To improve readability, labels have been added only to nodes present in at least half the plotted samples. Taxa that could not be confidently identified are indicated by an asterisk (*).

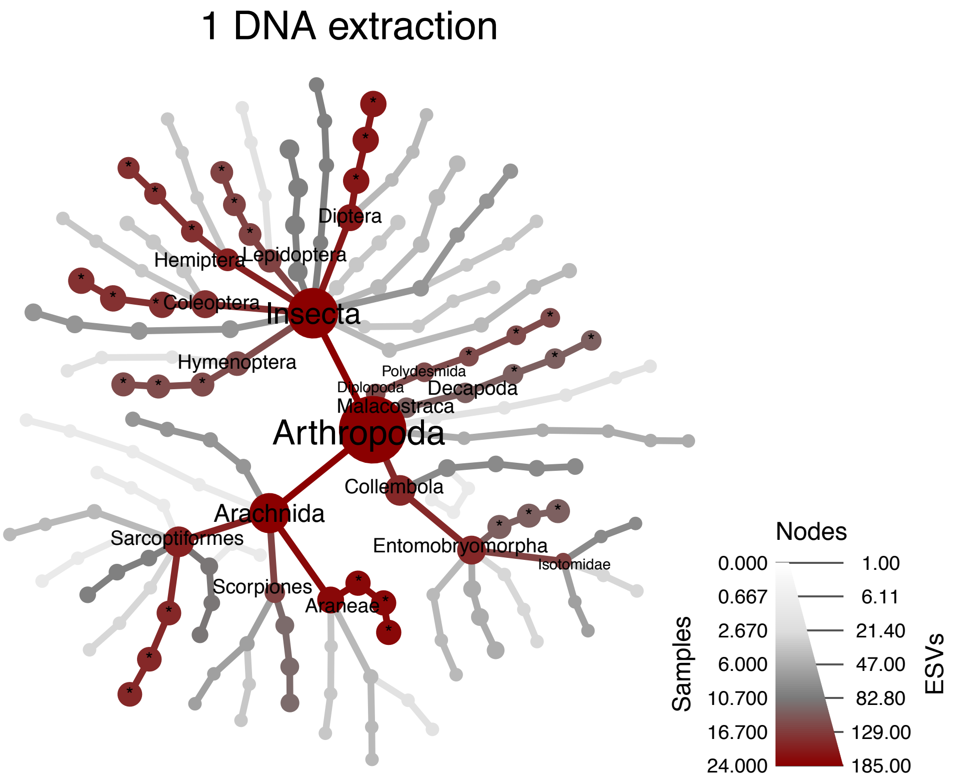
1 DNA extraction
Diptera
Hemiptera
Lepidoptera
Coleoptera
Insecta
Hymenoptera
Polydesmida
Diplopoda
Decapoda
Malacostraca
Arthropoda
Collembola
Arachnida
Sarcoptiformes
Entomobryomorpha
Isotomidae
Scorpiones
Araneae
Nodes
Samples
ESVs
0.000
0.667
2.670
6.000
10.700
16.700
24.000
1.00
6.11
21.40
47.00
82.80
129.00
185.00

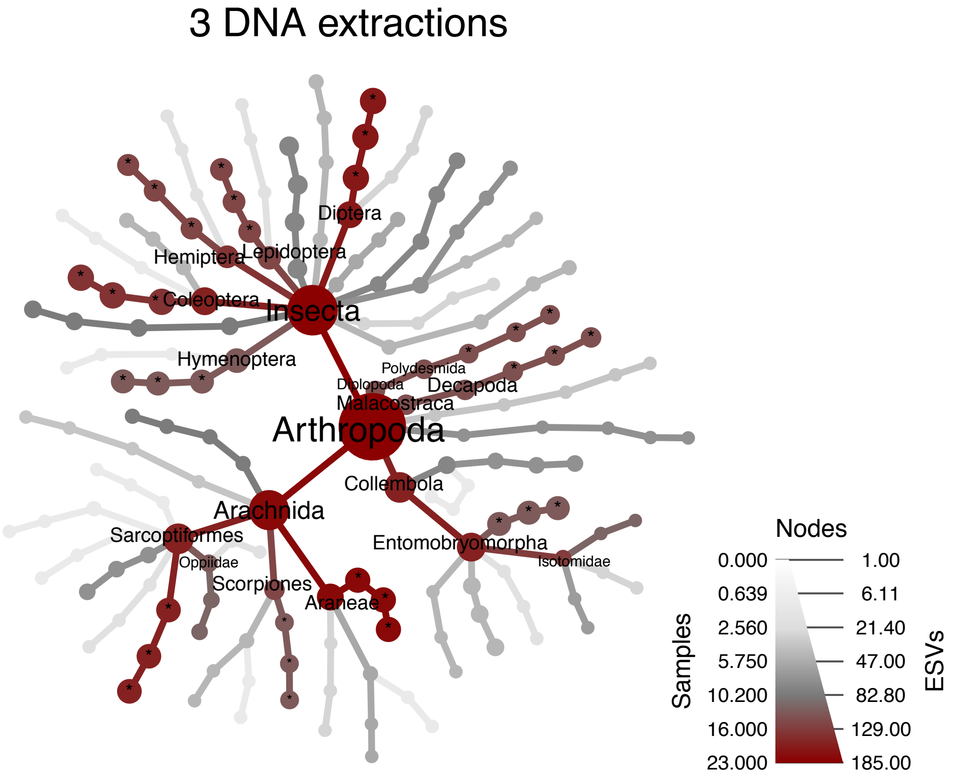
3 DNA extractions
Diptera
Hemiptera
Lepidoptera
Coleoptera
Insecta
Hymenoptera
Polydesmida
Diplopoda
Decapoda
Malacostraca
Arthropoda
Collembola
Arachnida
Sarcoptiformes
Oppiidae
Entomobryomorpha
Isotomidae
Scorpiones
Araneae
Nodes
Samples
ESVs
0.000
0.639
2.560
5.750
10.200
16.000
23.000
1.00
6.11
21.40
47.00
82.80
129.00
185.00

Fig S9. Site indicator taxa are largely recovered from the bryophyte and organic layers. Heat trees comprised of all the site indicator ESVs detected for each site-layer combination. For the 1C1E and 1C3E methods, we subsampled down to the same 4 grid coordinates for a balanced comparison across 7 methods (1C1E, 1C3E, 2C3E, 4C3E, 6C3E, 8C3E, 915C3E). In each heat tree, color indicates the number of samples where each taxon was detected; text and node size represent the number of site indicator ESVs in each taxon. To improve readability, labels have been added only to nodes present in at least half the plotted samples. Taxa that could not be confidently identified are indicated by an asterisk (*).

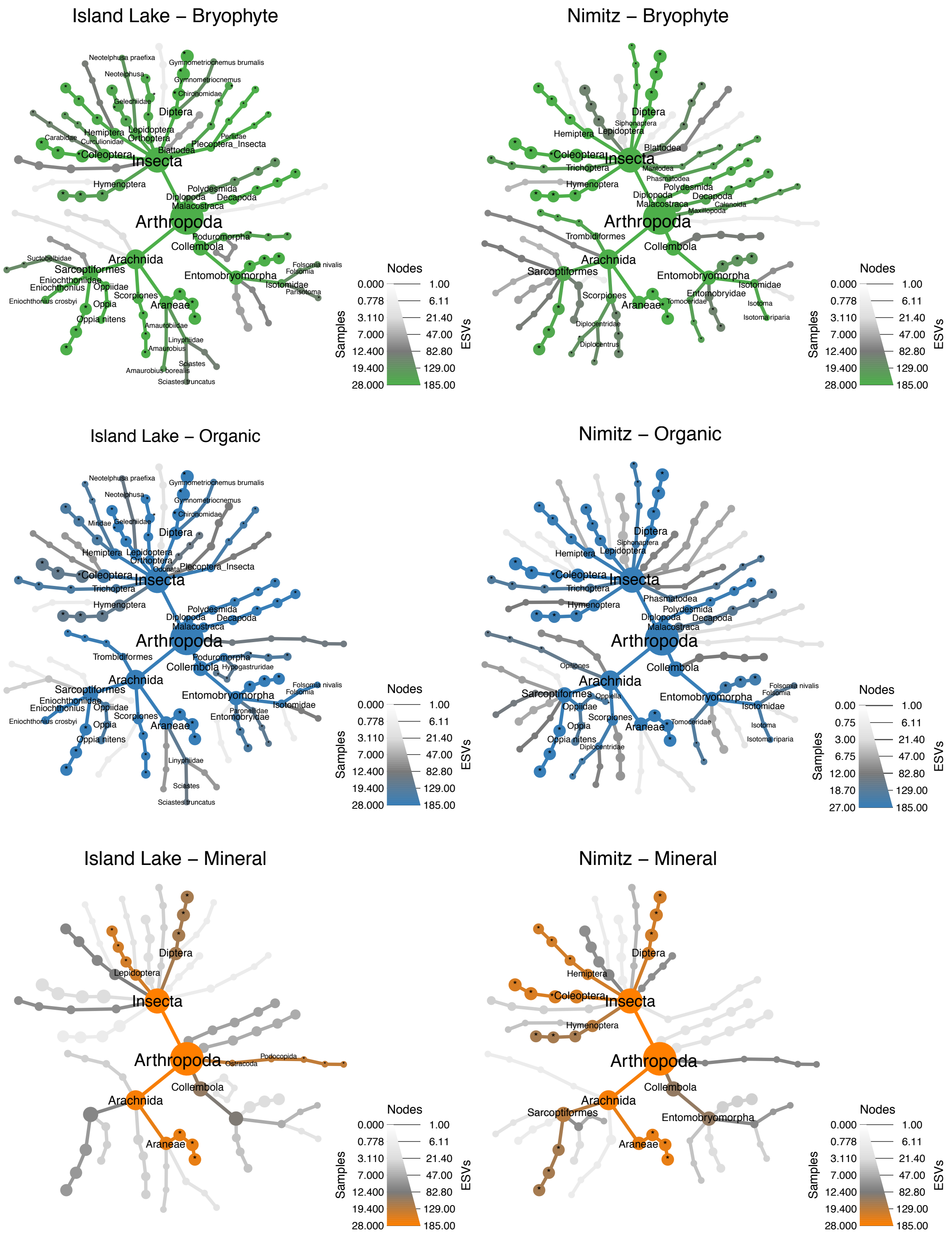

Island Lake – Bryophyte
Nimitz – Bryophyte
Island Lake – Organic
Nimitz – Organic
Island Lake – Mineral
Nimitz – Mineral
Arthropoda
Insecta
Arachnida
Collembola
Diptera
Hemiptera
Lepidoptera
Coleoptera
Hymenoptera
Trichoptera
Blattodea
Orthoptera
Plecoptera_Insecta
Polydesmida
Diplopoda
Decapoda
Malacostraca
Poduromorpha
Entomobryomorpha
Isotomidae
Entomobryidae
Sarcoptiformes
Trombidiformes
Oppiidae
Oppia
Oppia nitens
Scorpiones
Araneae
Linyphiidae
Sciastes
Sciastes truncatus
Folsomia nivalis
Folsomia
Nodes
Samples
ESVs
0.000
0.778
3.110
7.000
12.400
19.400
28.000
1.00
6.11
21.40
47.00
82.80
129.00
185.00
